# Peak width of skeletonized mean diffusivity might be a better neuroimage marker for cognitive functions in Cerebral Autosomal Dominant Arteriopathy with Subcortical Infarcts and Leukoencephalopathy (CADASIL)

**DOI:** 10.1002/alz.088185

**Published:** 2025-01-09

**Authors:** Wei‐Ju Lee, Fan Huang, Shi‐Ming Wang, Hung‐Chieh Chen, Yi‐Ming Chen, Hsu‐Wen Huang, Chih‐Mao Huang

**Affiliations:** ^1^ Taichung Veterans General Hospital, Taichung Taiwan; ^2^ National Yang Ming Chiao Tung University, Taipei Taiwan; ^3^ National Chung Shing University, Taichung Taiwan; ^4^ National Yang Ming Chiao Tung University, Hsinchu Taiwan; ^5^ National Chung Hsing University, Taichung Taiwan; ^6^ National Health Research Institutes, Yunlin Taiwan

## Abstract

**Background:**

Cerebral Autosomal Dominant Arteriopathy with Subcortical Infarcts and Leukoencephalopathy (CADASIL) is a monogenic subcortical ischemic vascular dementia (SIVD) caused NOTCH3 gene mutations. Many different neuroimaging methods have been used to investigate the association between neuroimaging changes and clinical symptoms. We investigated the correlation between peak width of skeletonized mean diffusivity (PSMD) values and cognitive functions in preclinical CADASIL patients without a history of stroke or dementia, comparing them to white matter hyperintensities (WMH) volumes.

**Method:**

This study recruited NOTCH3 R544C mutation carriers (n=63) without a history of stroke or dementia and age‐ and sex‐matched non‐carriers (n=35) in Taichung Veteran General Hospital. Brain MRI including T1, Fluid‐Attenuated Inversion Recovery (FLAIR), and diffusion tensor imaging (DTI) data were acquired on 1.5T MRI and neuropsychological assessments were used to evaluate the cognition functions for all participants. The domain‐specific composite Z‐scores were calculated to represent memory, executive function, processing speed, and visuospatial function. We examined the relationships between domain‐specific cognitive functions and both PSMD values and WMH volumes.

**Result:**

In this study, the data from 65 participants carrying NOTCH3 R544C mutation and 35 non‐carriers were analyzed. Mutation carriers have more severe WMH (p < 0.001) and longer PSMD values (p = 0.011) compared to non‐carriers. After adjusting for age, sex, and years of education using partial correlation, the PSMD values are associated with processing speed (r = ‐0.252, p = 0.048) and visuospatial functions (r = ‐0.275, p = 0.03). The correlations between WMH volume and the cognitive functions were not statistically significant.

**Conclusion:**

In summary, the PSMD value might be a better imaging biomarker for the cognitive functions in preclinical CADASIL patients and represent the early subcortical cerebrovascular damage compared to WMH volumes.

